# What paediatricians need to know about modern urologic management of vesicoureteral reflux

**DOI:** 10.3389/fped.2025.1607019

**Published:** 2025-06-26

**Authors:** Jeanne Goulin, Delphine Demède, Bruno Ranchin, Mélodie Mosca, Aurélie De-Mul, Valeska Bidault Jourdainne

**Affiliations:** ^1^ Department of Pediatric Urovisceral, Thoracic and Transplantation Surgery, HFME, Civil Hospices of Lyon, Lyon, France; ^2^ MARVU Reference Center for Rare Congenital Malformations of Urinary Tract, Lyon, France; ^3^ Claude Bernard Lyon 1 University, Lyon, France; ^4^ Department Pediatric Nephrology, Dermatology and Rheumatology, HFME, Civil Hospices of Lyon, Lyon, France; ^5^ Inserm U1208 Stem Cells and Brain Research Institute, Bron, France

**Keywords:** vesicoureteral reflux, reflux nephropathy, paediatric urology, ureteral reimplantation, urological management

## Abstract

Vesicoureteral reflux (VUR) is a common urological disorder in children, and its prevalence is difficult to determine, as many VUR cases remain asymptomatic. VUR is considered nearly physiological during infancy and often resolves spontaneously within the first few years of life. Most patients present with low-grade VUR, which is thought to be caused by an insufficient intramural course of the ureter in the bladder wall, a condition that tends to improve as the child grows and develops. The higher the grade of VUR , the lower the probability of spontaneous resolution of this condition during early childhood. Knowledge of the pathophysiology of VUR and renal scarring has evolved over the past decades. While surgical correction of VUR is thus often discussed, it is ultimately reserved for very selected cases, mainly when high-grade VUR persists despite the correction of voiding disorders in toilet-trained children and is associated with recurrent febrile UTI. European and other international pediatric urology societies have published guidelines for VUR management in children. With minimally invasive surgery becoming increasingly common among pediatric urologists, treatment modalities for VUR have evolved significantly, and endoscopic, laparoscopic, and robotic-assisted procedures now play a central role in surgical management. The recently updated ESPU/EAU guidelines are considered as a reference for pediatric urologists across Europe. In this review, we examine the recent literature and these guidelines to provide pediatricians with up-to-date data on VUR pathophysiology, its renal consequences, and current approaches to urological management.

## Pathophysiology of primary VUR and reflux nephropathy

### Primary and secondary VUR

Vesicoureteral reflux is defined as the backward flow of urine from the urinary bladder into one or both ureters, the renal pelvises, or both. It results from an incompetent uretero-vesical junction and may be classified as secondary, due to voiding disorders and bladder dysfunction (secondary VUR), or primary, occurring in the absence of any bladder abnormality (1–3). Normally, progressive distension of the bladder during the filling phase increases compression of the intramural and submucosal segment of the ureter against the detrusor muscle, thus preventing the retrograde flow of urine into the distal ureter. However, the effectiveness of this valve function depends on the length of the distal ureter located within the bladder wall: a shorter intramural–submucosal segment increases the risk of VUR (4). Its severity is graded in different ways. The International Reflux Study Committee grading system is the most widely used in children (5): reflux grades increase from I to V, according to retrograde and voiding cystourethrography imaging. Grade I indicates urine reflux into the lower part of the ureter, grades II to IV indicate reflux reaching the pelvis with progressive dilation of the collecting system (grades III and IV), and grade V indicates significant dilation and kinking of the ureter, dilation of the collecting system, papillary impressions no longer visible in most calyces, or intraparenchymal reflux (6, 7). VUR in children has been considered for several decades as an important risk factor for febrile urinary tract infections (UTI) and postinfectious scarring. Primary VUR is common in patients with a history of febrile UTI, with a prevalence of almost 40% in such cases, but also in nearly 20% of patients with antenatally diagnosed hydronephrosis (8). In the general population, VUR is probably present in 1% of children. Its incidence increases in specific populations, such as siblings of patients with VUR (14%–27%) or children of parents with known VUR (36%) (9–16). In cases of lower urinary tract disorders (LUTDs), secondary VUR occurs in half of patients (17); conversely, about one-third of patients with VUR have an underlying LUTD, mostly dysfunctional voiding (18). LUTDs are risk factors for persistent RVU, renal scarring, and reflux nephropathy.

### Renal scarring and reflux nephropathy

VUR is associated with renal scarring and renal dysplasia, which are major causes of chronic kidney disease, end-stage kidney disease, and hypertension in children (19–21). Reflux nephropathy (RN) may result from urinary tract infections (UTIs) caused by VUR, but it can also arise from renal dysplasia developed during fetal life and early infancy and is found to be associated with VUR, even in the absence of any UTI (22, 23). Thus, RN can be categorized as either congenital or primary lesions due to impaired renal development during fetal and neonatal life, leading to renal dysplasia or secondary acquired defects, due to renal scarring following UTI. Renal dysplasia, found in up to 29% of infants before 6 months of life with non-symptomatic VUR, may develop as a result of VUR and urine backward flow from the bladder, although this hypothesis is still not proven (24). But it could also be the hallmark of an impaired development of the ureteral bud and induction of the metanephric differentiation, leading to dysplastic nephrons, as exposed by Mackie and Stephens (25, 26). Specific risk factors for renal scarring following acute pyelonephritis in VUR patients classically include higher grade of VUR, voiding dysfunction or elimination disorders, recurrent pyelonephritis, or delayed start of antibiotic therapy (9). Recently, several studies have addressed this question and reduced the risk factors for recurrent febrile UTI and reduced differential split renal function at baseline evaluation and older age (after 1 year, and even more after toilet training) (27–30). In the absence of UTI, as long as urine remains sterile, there is no proven causality link between VUR and renal scarring, except for one study by Goren et al. that established a correlation between detrusor pressure at reflux onset and new renal scars in the absence of fUTI (31). Recently, several studies (32–37) have emphasized the role of intrarenal reflux (IRR) on reflux nephropathy and renal growth, suggesting that it may have been underdiagnosed in older studies due to its fleeting nature during bladder filling and emptying. IRR occurs more frequently in younger patients under 2 years with high-grade VUR. According to these studies, IRR would be responsible for bacterial penetration deep in the renal parenchyma combined with focal parenchymal ischemia in response to elevated urine pressure, causing breakthrough of new febrile UTI, renal scarring, and even renal growth impairment (33, 35–38).

Overall, 10%–20% of patients with a focal uptake defect on a radionuclide scan will present with hypertension or end-stage renal disease (39, 40).

## Diagnosing and exploring VUR in children

### Clinical presentations of VUR

Two clinical presentations of VUR have been classically described in the literature, based on gender segregation of the disease. The first group includes male infants, diagnosed before 2 years of age, often presenting with a history of antenatal hydronephrosis. These boys are more likely to present with high grade VUR, an abnormal DMSA scan and initial renal ultrasound, and are more likely to require surgery due to less spontaneous VUR resolution rate under conservative management. On the contrary, girls in the second group are diagnosed at an older age, commonly after toilet-training, with lower grades of VUR and less likely to be associated with focal defects on an initial DMSA scan. They are, however, at higher risk of recurrent febrile UTI and more often present with bladder bowel dysfunction. The management of this group relies primarily on elimination disorders resolution and continuous antibiotic prophylaxis, but these girls should be regularly followed due to the risk of renal scarring secondary to febrile UTI (41, 42).

### Voiding cystourethrography

Voiding and retrograde cystourethrography (VCUG) is the gold standard imaging test for the diagnosis and staging of VUR in children (43). VUR severity was well defined in 1985 by the International Reflux Study Committee Grading System in Children (5), according uniformization of VUR staging between low (grades 1 and 2) and high grade VUR (grade 3, 4 and 5). VCUG also assesses other urinary tract parameters, such as active or passive VUR, the bladder wall aspect during filling and voiding phases, the voiding profile of the bladder neck and the urethra, and even post-voiding residue. However, interrater reliability for VUR grading according to VCUG is quite low (0.53–0.59) and depends on rater quality and experience (44, 45). Recently, machine learning and deep learning have been introduced in VCUG interpretation to improve reliability of VUR detection and grading, with promising results (46). Noteworthy, to limit radiation exposure in young children and avoid discomfort due to urethral catheterization (47), other cystogram modalities are emerging, such as contrast-enhanced voiding urosonography or direct radionuclide cystography. Contrast-enhanced voiding cystosonography (ceVUS) was developed in the 1990s, injecting either galactose or air bubbles infused with saline directly into the bladder through an urethral catheter. VUR can be visualized directly with standard US probes used for urinary tract exploration. It displays good to excellent correlation with VCUG for VUR detection, sometimes upgrading low-grade reflux but rarely missing the diagnosis (48–54). It could be used for VUR follow up or VUR diagnosis in girls or high-risk patients, for example after kidney transplantation (48, 53). CeVUS displays a particularly good sensitivity for IRR detection, offering live visualization of the contrast agent flowing back from the calyces in the tubulo-interstitial space. Recent publications demonstrate that IRR could be detected in 12%–62% of all VUR cases with this technique, far beyond the 1%–11% detected by standard VCUG (36, 38, 53). As IRR may be an important predictive factor of renal scarring, some experts now recommend using this modality prior to VCUG for VUR diagnosis (36, 38, 51, 53), although it remains as invasive as VCUG (urinary catheter placement), requires trained imaging specialists, and sometimes misses low grade VUR (51, 53–55).

Other imaging modalities (magnetic resonance VCUG, nuclear cystography, and direct radionuclide cystography) are still rarely used, notably because some of them still require urethral catheterization and radiation and need further validation to replace VCUG in general practice (56–58).

### Bottom-up or top-down approach?

For several decades, the standard approach to assess children for potential VUR was to directly assess VUR by voiding retrograde cystourethrography (VCUG) after renal ultrasound (US). Patients eligible for VCUG either had antenatal ureteral and/or pelvic dilation confirmed after birth by renal US or were explored after treatment of febrile UTI (fUTI) with or without normal renal US. In 2011, the Subcommittee on Urinary Tract Infections of the American Academic of Pediatrics recommended limiting VCUG investigation after at least two episodes of fUTI or after the first episode of fUTI in cases of abnormal renal US, such as pelvic or ureteral dilation, renal scars, or bladder wall abnormality (47). This approach, named the bottom-up approach after fUTI, offers the advantage of confirming the presence or absence of VUR of any grade but with the risk of discomfort due to urethral catheterization or induced pyelonephritis in patients who usually do not have high grade VUR or renal damage but do have a high probability of spontaneous resolution of VUR (59). Thus, another diagnostic strategy emerged in the 2000s for the exploration of patients after fUTI based on analysis of the risk of clinically significant VUR in cases of renal parenchymal damage or renal scarring (60, 61). This top-down approach favors DMSA scans to VCUG as a first-line exploration right after the detection of fUTI in patients, initially without performing renal US because of the low detection rate of significant abnormalities with this scan (62, 63). The DMSA scan is believed to discriminate patients at risk of long-term renal scarring by identifying acute inflammation spots in renal parenchyma. With this approach, VCUG and renal US are only proposed if there are significant parenchymal inflammatory changes or renal scars on the DMSA scan to detect intermediate- to high-grade VUR. It has the advantage of limiting VCUG exploration to patients with a high probability of clinically relevant VUR, as it has been proven that this approach misses only VUR of low or intermediate grades that have no further clinical significance and might resolve spontaneously within five years (60). This approach also reduces antibiotic prophylaxis exposure in children only recommended in cases of confirmed high-grade VUR (63). However, when comparing all VUR diagnosis and modern management approaches, neither the top-down nor the bottom-up approach succeeded in demonstrating diagnostic superiority to one another: the top-down approach displays the best sensitivity for renal scarring, but economic and safety studies have demonstrated higher costs and radiation levels than others (59, 61). As for treatment and renal prognosis, a recent study tried to compare bottom-up and top-down approaches regardless of patient age. This study demonstrated that the top-down approach was associated with a slightly higher rate of recurrent fUTI but with less continuous antibioprophylaxis (CAP) exposure in children and much lower rates of VCUG performed; this study failed to demonstrate any difference in new renal scarring between the two approaches (64). From this perspective, ceVUS may represent a promising VUR diagnostic tool, displaying the same sensitivity for VUR detection as VCUG and even better performances for IRR detection, without any radiation risk for the patient (36, 51). As a consensus, recently updated EAU/ESPU guidelines recommend performing renal US exploration after fUTI in all children. Children presenting with an abnormal renal US scan, or before one year of age, have a higher risk of high-grade VUR or urinary tract malformation and should then undergo VCUG for VUR detection and then a DMSA scan if VUR is proven. The top-down approach can be considered in children older than one year, with a high negative predictive value of high-grade VUR when combined with a normal renal US (40, 65). In current practice, many pediatric nephrologists choose to limit radiation exposure or infectious risk of invasive explorations like DMSA scan or VCUG and instead try conservative management of VUR after only renal ultrasound. They do not apply the top-down approach because of the mixed results of high-grade VUR detection based on DMSA scans (93% sensitivity but 44% specificity) (66), and patients are only offered VCUG if they fail this first-line conservative management. However, this attitude has not been retained in the latest recommendations from the ESPU/EAU societies, probably because of the relatively low performances of renal US alone for high-grade VUR detection (59% sensitivity and 79% specificity) (66, 67).

### VUR impact on renal parenchyma

Renal parenchymal impairment developed during fetal life or after febrile UTI is classically assessed by a technetium 99m-Dimercaptosuccinic Acid scan (DMSA scan). Although renal US could detect renal parenchymal abnormalities like scars with excellent specificity, its sensitivity is too low to serve as a gold standard (67). Recent studies, however, underline the good correlation between IRR detected by ceVUS, high-grade VUR, and reflux nephropathy, providing additional arguments for the promotion of contrast-enhanced US imaging in VUR diagnosis (34, 36, 38, 55). Concerning renal nuclear imaging, there is no strong consensus on the minimal time interval that should be observed after a febrile UTI to explore renal scarring with strong reliability. Most experts in renal scintigraphy agree that DMSA scans show mostly permanent cortical defects after an interval of 3–6 months, which is sometimes difficult to achieve in cases of recurrent febrile UTI (4, 68). Experts also agree that DMSA scans can be performed at any age after one week of life with good reliability (68). As stated above, in case of symptomatic VUR, recurrent febrile UTI and reduced differential renal function are independent risks factors for renal scarring (27, 29, 30): around 30% of patients with intermediate- or high-grade VUR and a history of febrile UTI have renal scars on follow-up DMSA scans compared to less than 10% of asymptomatic refluxing patients (69). From the urological point of view, at least one DMSA scan should be performed during the evaluation or follow-up of a patient with VUR to assess VUR impact on renal parenchyma and guide its management—at diagnosis in top-down approach cases or after VCUG in other situations (40).

## Modern urological management of VUR

The aim of VUR management is to prevent the recurrence of febrile UTI, avoid renal scarring, and preserve kidney function. This management is primarily based on a conservative or medical approach, associating watchful waiting, continuous antibiotic prophylaxis (CAP) bladder bowel dysfunction (BBD) correction, and, for some pediatric nephrologists, medical management of the prepuce in boys. In case of persistent symptomatic VUR or renal scarring despite this non-surgical management, more “aggressive” treatment may be indicated, relying on VUR corrective surgery. Treatment options must be individually tailored, and there is still controversy between specialists on the best management practice, particularly regarding the timing of surgery.

### Non-surgical therapeutic options

#### Continuous antimicrobial prophylaxis

Continuous antimicrobial prophylaxis has long been prescribed in cases of VUR or upper urinary tract dilatation, regardless of VUR grade, with low evidence of its benefit on infectious recurrence and long-term renal function prognosis (70). Recent studies have addressed this specific question, with the goal to produce strong scientific data on CAP benefit, such as the Randomized Intervention for Vesico-Ureteral Reflux (RIVUR) study or the PREDICT study (24, 71, 72). Based on the RIVUR study, the first analysis published in 2014 concluded that all grades of VUR would benefit from CAP to reduce breakthrough febrile UTIs. These results were included in a meta-analysis published in 2015, the results of which initially concluded a significant reduction of fUTI in high-grade VUR only but were amended and finally supported CAP in all VUR grades (73). Reanalysis of the RIVUR study was finally conducted in 2018 by Wang et al. It concluded this time that specific groups had a higher risk of recurrent fUTI but not more frequent renal scarring; these groups included uncircumcised boys and cases with bilateral VUR or associated BBD (74). In the absence of a first episode of fUTI, the PREDICT study demonstrated the ability of CAP to reduce breakthrough fUTI in high-grade RVU (grades III or IV), with seven patients treated over two years to avoid pyelonephritis (24). Recent studies on ceVUS may also suggest that patients with identified IRR would benefit from prolonged CAP due to higher risk of renal scarring (36, 53). Molecules used for prophylaxis include trimethoprim/sulfamethoxazole, nitrofurantoin, or amoxicillin. Trimethoprim should be avoided before 6 weeks of age due to the risk of liver damage, as well as in cases of severe renal failure, but it is the most frequently prescribed, at a median dose of 10 mg/kg/day of trimethoprim. Selection of trimethoprim-resistant microbiota, particularly *Escherischia coli* species, is described in some studies (75, 76), but it seems to be related to fewer recurrences of febrile UTI. Parental adherence to treatment is a major concern in such chronic prophylactic treatment, and Craig et al. recorded up to 30% treatment discontinuation at one-year follow-up of children with primary VUR (77).

#### Circumcision

Circumcision has been proven to reduce the risk of recurrent febrile UTI in male infants since the late 1990s, even in cases of antenatally diagnosed dilating VUR without previous breakthrough fUTI (78–82). A systematic review with meta-analysis conducted in 2005 observed a significant reduction of febrile UTI in circumcised boys (OR 0.13) but, considering the complication rate of circumcision, it concluded that benefits occur only in children at high risk of UTI (history of past UTI or high grade VUR) (83). Even a retractable foreskin which allows easy visualization of the glans is associated with a lower risk of recurrent fUTI compared to severe phimosis in young children (84–86). Holzman et al. published a hazard ratio (HR) of 8.5 of recurrent febrile UTI in boys with severe phimosis compared to circumcised boys or boys with mild phimosis (at least partially retractable foreskin with visualization of the glans meatus). This protective effect would be linked to the reduction of bacterial colonies around the glans and the urethral meatus. Güçük et al. demonstrated that prophylaxis itself did not modify the periurethral flora but that circumcision did, with a lower periurethral bacterial load (87). Moreover, circumcision changes the composition of the bacterial flora, with more epidermal germs and less classical uropathogens, whether prophylaxis is added or not (87, 88).

#### Bladder and bowel dysfunction management

Bladder and bowel dysfunction (BBD) is characterized by the presence of one or several different dysfunctional elimination syndromes: infrequent voiding, constipation, dysfunctional voiding syndrome, or overactive bladder (known previously as bladder instability) (89). BBD is common in children with primary VUR and should be explored in every toilet-trained patient with VUR or recurrent fUTI, since it is an independent risk factor for VUR persistence, recurrent fUTI, renal scarring, and failure of VUR surgical correction (18, 27, 90–93). The most concerning patterns are incomplete voiding with postvoiding residue and other dysfunctional voiding symptoms, which negatively correlate with VUR resolution and treatment success (18, 91, 94–97). BBD rehabilitation is based on standard urotherapy: timed voiding every two to three hours, correction of the child's voiding position, water intake increase, and bowel management with laxatives, with the objective of at least one bowel movement each day, with smooth and easily defecated stools. Parasacral Transcutaneous Electrical Stimulation (TENS) is emerging as a new treatment option for BBD associated with urophysiotherapy (98).

### Surgical options for VUR correction

#### Sub-ureteric endoscopic injection of bulking agent

Introduced in the 1980s, sub-ureteric injection of a bulking agent gained progressive popularity among pediatric urologists because of its overall mean efficacy of 85% after one or repeated injections and its simple administration through cystoscopy on a day surgery basis (99). The lower the VUR grade is, the higher the VUR resolution rate after one subureteric injection (78.5% in grade 1 or 2 vs. 51% in grade 5) (100). Agents used were initially non-absorbable synthetic agents, like polytetrafluoroethylene (PTFE or Teflon) or polydimethylsiloxane, which is silicone-based (Macroplastique®, Congentix Medical, Orangeburg, NY, USA) (101, 102). These agents had the disadvantage of generating a chronic inflammatory response with a granuloma formation around the injection site, posing a risk of secondary ureteral obstruction, and they were also at risk of migration due to the small size of the particles injected (4–100 µm). Biocompatible agents were thereafter developed, and one has become the most adopted bulking agent to date since its approval by the Food and Drug Administration in the USA: dextranomer/ hyaluronic acid (Dx/HA, Deflux®, Salix Pharmaceuticals, NJ, USA). Most recently, a systematic review and meta-analysis indicated that a synthetic non-biodegradable substance, named Polyacrylate Polyalcohol Copolymer (PPC) (Vantris®, Promedon, Cordoba, Argentina), proved similar to better VUR resolution rates at short- and long-term follow-up compared to Deflux®. However, PPC injection was at higher risk of ureterovesical junction (UVJ) obstruction, sometimes occurring several months after injection due to inflammation and fibrosis of the UVJ and requiring ureterovesical reimplantation (103). Techniques of endoscopic injection have also been a source of debate for years. The first described was the subureteric injection, or STING technique (99), in which the bulking agent is injected under the mucosa by needle puncture 2–3 millimeters under the ureteral meatus. The Hydrodistension Implantation Technique (HIT) was then developed, with the introduction of the needle in the mucosa of the ureteral channel and injection of the bulking agent into the distal ureter. Finally, the double HIT technique became the most used technique for endoscopic injection in the USA (104), with double puncture of the ureteral mucosa in the distal ureter (105). A recent systematic review and meta-analysis indicated that HIT had a higher VUR resolution rate (82.5%) than standard STING procedure with Dx/HA (72.4%) [pooled odds ratio (OR) = 0.54; 95% confidence interval 0.42–0.69; *p* < 0.0001; I2 = 8%] but did not conclude the superiority of HIT since no significant difference was found between the two techniques for the need of re-injection (106). The double HIT injection seems to have higher success rates than STING and HIT, but few studies have addressed this question (104, 107).

#### Ureteral reimplantation

Ureteral reimplantation is the gold standard treatment for VUR, first described in the 1950s (108). It aims to create a new submucosal channel for the distal ureter, measuring at least 4 times the ureteral diameter (Paquin's law) (109), in order to obtain a passive flap valve mechanism with satisfactory collapse of the distal part of the ureter when intravesical pressure increases. This reimplantation is associated eventually with the creation of a new ureteral meatus (neo-meatus) at the same time. Multiple techniques have been described over the years, with an excellent success rate of 95%–98% and low complication rate (110, 111). These techniques are sorted between intravesical and extravesical approaches, depending on bladder surgical opening or not. The intravesical reimplantation developed by Cohen is the most frequently performed (112). This is an infra-hiatal reimplantation, since it consists of the intravesical dissection of the refluxing meatus and distal ureter, and then reimplantation with a submucosal channel across the trigone, downstream from the native ureteral meatus, toward the opposite ureter (112). This technique has excellent results but makes upper tract access difficult, for example during endoscopic procedures in cases of urolithiasis (113, 114). The main alternative to this technique is the suprahiatal Politano Lead Better ureteroneocystostomy, in which a new entry point for the ureter in the detrusor muscle is created, with a submucosal channel upstream from the native ureteral meatus in the bladder (115). When extravesical reimplantation is favored, the technique developed by Lich and Gregoir is almost the only one used in children, consisting of separating muscle fibers of the detrusor just upstream from the ureterovesical junction, placing the ureter in the submucosal channel created, and suturing the detrusor above the distal part of the ureter (116, 117). Results of this technique are overall satisfying and similar to those of Cohen's reimplantation, with lower hospital stay and bladder catheter duration (118). However, this technique is less applicable to bilateral cases due to the extravesical dissection of the bladder trigone that might trigger urinary retention, contrary to Cohen's technique, which displays the same results in uni- or bilateral VUR (119).

Open surgery has long been the only option available for these reimplantation techniques, with longer hospital stays and bladder catheter duration than endoscopic treatment (120). But, over the last two decades, minimal invasive surgery has become more popular in expert urological teams, and “radical” VUR surgical treatments have regained interest through vesicoscopic and laparoscopic approaches (121–123). The vesicoscopic approach offers the advantage of smaller scars and lower post-operative pain, with similar success rate to the open Cohen technique, but was only adopted in a small number of expert teams due to the steep learning curve of the technique (121, 122, 124). Laparoscopic extravesical vesicoureteral reimplantation (LEVUR) and, more recently, robotic-assisted extravesical vesicoureteral reimplantation (RALUR) offer good minimally invasive alternatives to open Lich Gregoir reimplantation, with excellent VUR resolution rates of 96% and 93% respectively in recent literature. LEVUR and RALUR display similar complication rates of 5.4% and 6.6%, respectively, notably post-operative urinary retention in bilateral cases (119, 125). Acute urinary retention mostly occurs after extended retrovesical dissection at the trigonal level in bilateral VUR cases but fortunately is mostly transient during the first post-operative days (126). Although the development of nerve-sparing techniques mean shorter hospital stays and indwelling catheter duration for extravesical robotic-assisted reimplantation (127, 128), due to longer operative time and overall higher costs, RALUR has not supplanted LEVUR or the open Lich Gregoir technique in recent literature or surgical recommendations (40, 119, 125, 129).

Finally, in cases of recurrent fUTI and poorly functioning renal unit (Split Renal Function <10%), nephrectomy can be considered.

#### Management strategies

**Figure 1:**
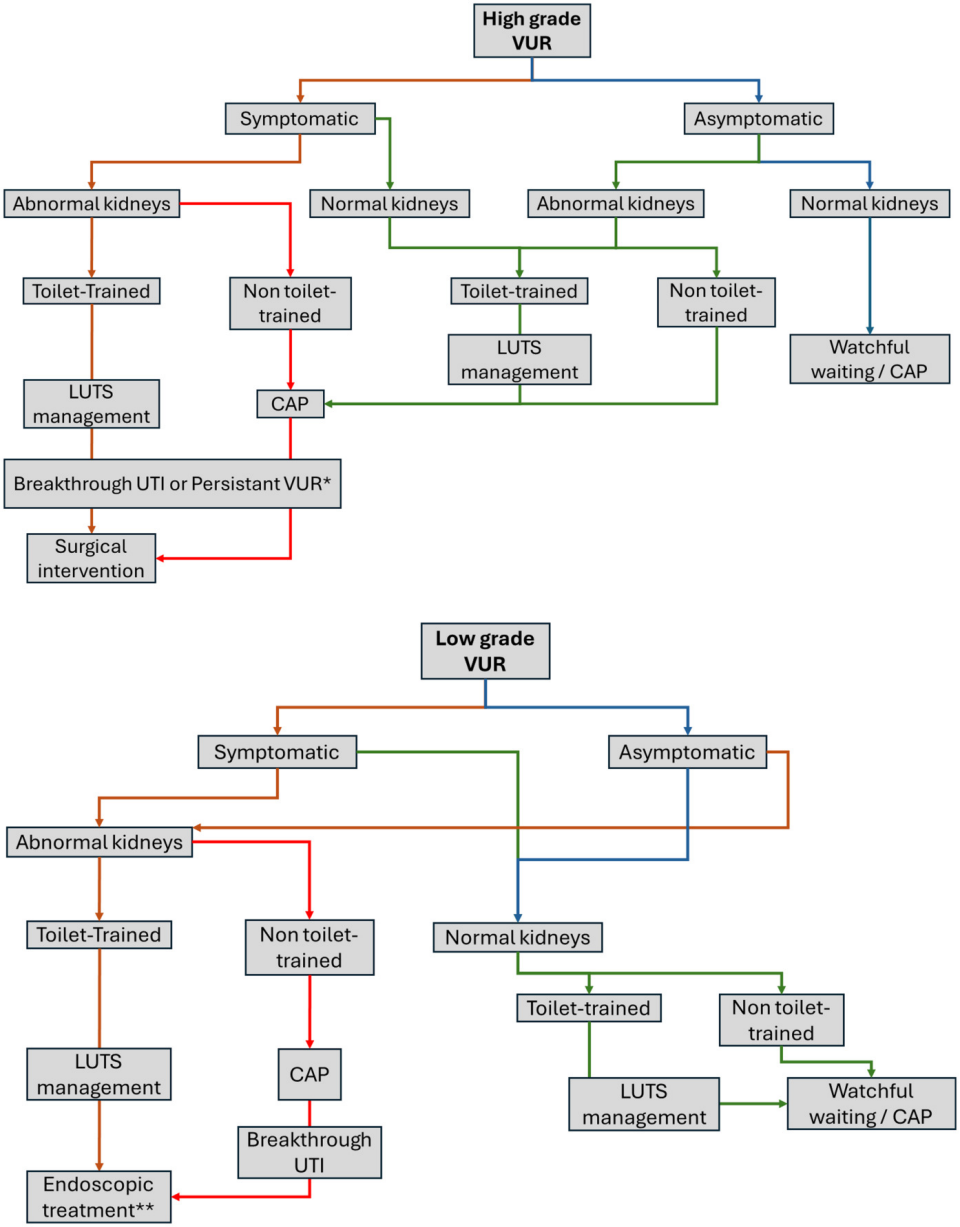
VUR management as proposed by EAU/ESPU 2024 recommendations (40). VUR, vesico-ureteral reflux; LUTS, lower urinary tract symptoms; CAP, continuous antimicrobial prophylaxis; UTI, urinary tract infection. *No consensus in selected cases after multidisciplinary team discussion. **Controversial, first intention only after LUTD management and breakthrough UTI.

Since 2012, the European Association of Urology (EAU) and the European Society for Pediatric Urology (ESPU) have established recommendations for primary VUR management in children that are regularly revised to adjust to medical and surgical advances (7, 40, 43). This management relies on the stratification of the risk of recurrent fUTI, renal parenchymal scarring, and permanent renal function impairment due to VUR that underlie the indications for early intervention. This risk evaluation encompasses the clinical course (febrile and non-febrile UTI), the grade of reflux, ipsilateral renal function, bilaterality, bladder function, associated anomalies of the urinary tract, existing renal scars, and age of the patient. Recently, experts have underlined the importance of tailored individualized approaches for each patient based both on the risk evaluation of renal scarring and on personal and familial history and parental adherence to treatment. Actualized EAU/ESPU recommendations are summarized in Figure 1.

#### Conservative management

Conservative management of VUR relies on a combination of medical measures and on circumcision in boys. This approach is particularly important in infants, since they have the higher potential of spontaneous resolution of VUR while growing up. This management implies regular follow-up with renal imaging. Apart from renal US scans, monitoring of growth in height and weight, as well as blood pressure and biological workups for serum creatinine level and proteinuria should be part of the follow-up. There is no consensus in the literature on the optimal timing or frequency of such explorations, and follow-up should be adjusted for each patient, taking into account the individual risk of spontaneous resolution of VUR and new renal scarring. In a recent meta-analysis with a systematic review of the literature, Basiri et al. reviewed the factors classically involved in VUR resolution probability (130). They demonstrated that the only predictable factor for spontaneous resolution was VUR severity, with lower resolution probability the lower the VUR grade: 80% of spontaneous resolution for grade 1 contrary to 23% for grade 4. Gender or laterality did not seem to have any significant influence on this rate. Conservative management consists of watchful waiting, continuous antimicrobial prophylaxis (CAP) in selected cases, bladder and bowel dysfunction rehabilitation, and circumcision in boys. As stated above, CAP should be prescribed mainly in patients with high-grade VUR before toilet training or in the presence of BBD, regardless of VUR severity or patient's age. It could be interrupted after completion of toilet training or proof of VUR or BBD resolution in cases with no fUTI recurrence. In all other situations, it could be prescribed based on the practitioner's individual evaluation of the situation but with poor scientific proof of its benefit. Circumcision can be proposed to every boy with high-grade or symptomatic VUR, especially in infants aged less than one year. There is no consensus in the literature on the optimal timing and modalities of follow-up; they may include renal US every 6–12 months and regular DMSA scans depending on initial focal uptake defect on radionuclide renal scans. Repeated VCUG to assess VUR resolution seems unnecessary in the absence of recurrent fUTI. After breakthrough fUTI in the presence of CAP, treatment modalities should be reconsidered for VUR surgery.

#### Surgical therapy

In symptomatic cases of VUR, the classical indications for VUR surgical correction are recurrent fUTI despite CAP and/or circumcision in boys after toilet-training and BBD rehabilitation or new focal uptake defect on radionuclide scans . But treatment modalities should be tailored to each patient, and parental choice should be considered at each decisional step. There are practical scoring systems that may help practitioners decide between VUR treatment surveillance, CAP, or surgical treatment (like the Boston's Children Hospital VUR Resolution Rate Calculator or the iReflux Risk Calculator) (15, 131, 132). Endoscopic treatment is mostly indicated in low-grade VUR with good results (78% resolution rate) but, in cases of high-grade VUR, vesicoureteral reimplantation displays better resolution results than subureteric bulking agent injections. Open techniques remain the gold standard for high-grade VUR surgery, although minimally invasive approaches have become more widespread among pediatric surgeons (7, 40, 43). VUR surgery might be proposed in cases of persistent high-grade VUR after toilet-training but there is no consensus on the rationale, timing, or modalities of this surgery in this specific indication. In fact, no data on asymptomatic high-grade VUR are currently available to assess long-term renal and bladder outcomes; these cases should be carefully evaluated by multidisciplinary experienced teams before any surgical decision.

## Conclusion

Primary VUR urological management aims to preserve renal function by preventing future renal scarring due to recurrent fUTI. But VUR does not necessarily reflect a symptomatic disease and identifying patients at risk of renal deterioration is crucial. Voiding cystourethrography remains the gold standard diagnostic method to assess VUR and its severity. Renal involvement is established through DMSA radionuclide scans looking for cortical defects of fixation that represent reflux nephropathy. Most patients with primary VUR benefit from conservative management, which relies on medical measures while expecting spontaneous VUR resolution or improvement with growth, especially continuous antimicrobial prophylaxis in selected cases and rehabilitation for bladder and bowel disorders. When this approach does not prevent recurrent fUTI, surgical management is helpful. Controversies persist on the management of persistent high-grade VUR after growth and toilet-training and should be submitted to multidisciplinary team discussion if surgery is considered. Endoscopic treatment has increased in popularity due to its minimally invasive and easy application, with good results in low-grade VUR. Vesico-ureteral reimplantation is still the gold standard in high-grade cases, both by open or minimally invasive approaches.
